# Multimodal X-ray nano-spectromicroscopy analysis of chemically heterogeneous systems

**DOI:** 10.1093/mtomcs/mfac078

**Published:** 2022-10-08

**Authors:** Ajith Pattammattel, Ryan Tappero, Dmitri Gavrilov, Hongqiao Zhang, Paul Aronstein, Henry Jay Forman, Peggy A O'Day, Hanfei Yan, Yong S Chu

**Affiliations:** National Synchrotron Light Source II, Brookhaven National Laboratory, Upton, NY 11973, USA; National Synchrotron Light Source II, Brookhaven National Laboratory, Upton, NY 11973, USA; National Synchrotron Light Source II, Brookhaven National Laboratory, Upton, NY 11973, USA; Leonard Davis School of Gerontology, University of Southern California, Los Angeles, CA 90089, USA; Environmental Systems Graduate Program, University of California, Merced, CA 95343, USA; Leonard Davis School of Gerontology, University of Southern California, Los Angeles, CA 90089, USA; Environmental Systems Graduate Program, University of California, Merced, CA 95343, USA; Life and Environmental Sciences Department and the Sierra Nevada Research Institute, University of California, Merced, CA 95343, USA; National Synchrotron Light Source II, Brookhaven National Laboratory, Upton, NY 11973, USA; National Synchrotron Light Source II, Brookhaven National Laboratory, Upton, NY 11973, USA

**Keywords:** nanoprobe, xanes, data processing, machine learning, spectromicroscopy

## Abstract

Understanding the nanoscale chemical speciation of heterogeneous systems in their native environment is critical for several disciplines such as life and environmental sciences, biogeochemistry, and materials science. Synchrotron-based X-ray spectromicroscopy tools are widely used to understand the chemistry and morphology of complex material systems owing to their high penetration depth and sensitivity. The multidimensional (4D+) structure of spectromicroscopy data poses visualization and data-reduction challenges. This paper reports the strategies for the visualization and analysis of spectromicroscopy data. We created a new graphical user interface and data analysis platform named XMIDAS (X-ray multimodal image data analysis software) to visualize spectromicroscopy data from both image and spectrum representations. The interactive data analysis toolkit combined conventional analysis methods with well-established machine learning classification algorithms (e.g. nonnegative matrix factorization) for data reduction. The data visualization and analysis methodologies were then defined and optimized using a model particle aggregate with known chemical composition. Nanoprobe-based X-ray fluorescence (nano-XRF) and X-ray absorption near edge structure (nano-XANES) spectromicroscopy techniques were used to probe elemental and chemical state information of the aggregate sample. We illustrated the complete chemical speciation methodology of the model particle by using XMIDAS. Next, we demonstrated the application of this approach in detecting and characterizing nanoparticles associated with alveolar macrophages. Our multimodal approach combining nano-XRF, nano-XANES, and differential phase-contrast imaging efficiently visualizes the chemistry of localized nanostructure with the morphology. We believe that the optimized data-reduction strategies and tool development will facilitate the analysis of complex biological and environmental samples using X-ray spectromicroscopy techniques.

## Graphical Abstract

**Graphical Abstract:**
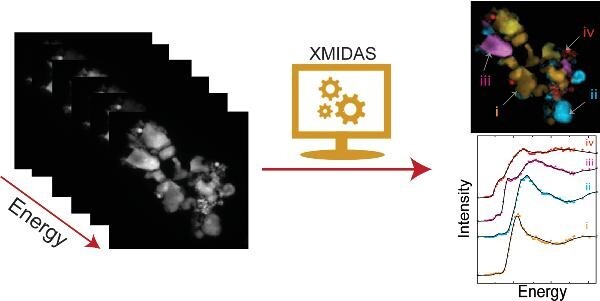
XMIDAS: A spectromicroscopy data analysis tool.

## Introduction

Chemical speciation of heterogeneous systems at nanometer spatial scales (nanoscale) is a challenging problem requiring high spatial resolution and high detection sensitivity. X-ray and electron-based spectromicroscopy tools are used to probe the electronic state of atoms and thereby map out the elemental and chemical distribution. Spectromicroscopy techniques are widely used to examine sample morphology or spatial heterogeneity, along with chemical speciation. Electron-based spectromicroscopy tools perform nanoscale analysis on heterogeneous systems by incorporating methods such as electron energy loss spectroscopy^1^ and energy-dispersive X-ray spectroscopy.^2^ For soft X-rays, scanning transmission X-ray microscopy (STXM) is a dominant spectromicroscopy technique utilizing absorption spectroscopy,^3^ while some instruments utilize fluorescence spectroscopy for a higher spectrum of the soft X-ray energy range.^4^ For hard X-rays, microprobes,^5^^,^^6^ and nanoprobes^7^^,^^8^ typically use X-ray fluorescence (XRF) detection for spectromicroscopy. In recent years, full-field-based chemical imaging using a transmission X-ray microscope demonstrated high-throughput nanoscale chemical imaging capability.^9^ However, this technique is unsuitable for imaging minority phases with trace-level concentrations. We recently reported a nanoscale chemical imaging technique at sub-50 nm resolution^10^ utilizing X-ray absorption near edge structure (XANES) through fluorescence detection. We implemented nano-XANES as a multimodal spectromicroscopy technique with simultaneous acquisition of fluorescence spectra and transmission data for phase-contrast imaging, using either transmission ptychography or differential phase-contrast (DPC) imaging.^11–13^ The multimodal nature of X-ray nanoprobe data can be used as a comprehensive characterization technique to provide a holistic understanding of the material system. Moreover, nano-XANES is sensitive to below ppm (parts per million) elemental concentrations in the hard X-ray regime. Thus, it is desirable for determining chemical speciation at trace level concentrations and ideally suited for biological and environmental studies. However, comprehensive analysis using this technique by nonexpert researchers is challenged by the lack of methodologies and computational tools to visualize and analyse the multimodal, cross-correlative datasets. For instance, nano-XANES data contain both structural and spectral information about the sample and thus require multiple image processing steps in combination with XANES spectrum processing to deduce meaningful chemical state maps. This paper illustrates how scanning mode nano-XRF, nano-XANES, and phase-contrast imaging can be combined and interpreted to identify nanoscale chemical speciation of particle mixtures in heterogeneous systems.

Scanning probe X-ray nano-spectromicroscopy is conducted by collecting XRF spectra while performing a 2D raster scanning of the sample at each energy point. The higher dimensional nature (e.g. 4D nano-XANES data) of spectromicroscopy data poses multiple data analysis and visualization challenges to extract meaningful information. Several software tools have been developed to address these challenges. Elemental analysis using XRF microscopy is a well-established field with a handful of analysis packages such as PyXRF,^14^ MAPS,^15^ SMAK,^16^ and PyMCA.^17^ Likewise, a few chemical mapping tools are also available but custom-made for transmission-based techniques.^18–20^ One conventional method of nano-XANES analysis fits the unknown spectrum with a linear least square combination of reference standard spectra. In addition, spectromicroscopy data can be decomposed using algorithms such as principal component analysis, nonnegative matrix factorization (NMF), and *k*-means clustering without prior knowledge of the sample.^18^ Application of these algorithms to nano-XANES analysis requires further optimization to demonstrate their accuracy in determining chemical speciation. Challenges in utilizing existing tools for analysing the nano-XANES data arise from the differences in spectral quality, normalization methods, background noise, and the need for correlating the XRF data with the additional data sets acquired simultaneously (i.e. ptychography or DPC). Thus, analysis of multimodal nano-XANES data^10^^,21^ presents opportunities for integrating dimensionality reduction algorithms. Therefore, the second thrust of this paper is to establish data analysis methodologies that use available machine learning algorithms to aid conventional methods of analysis. The user-friendly interface developed here provides an integrated platform for multimodal image data analysis using several independent workflows.

**Fig. 1:**
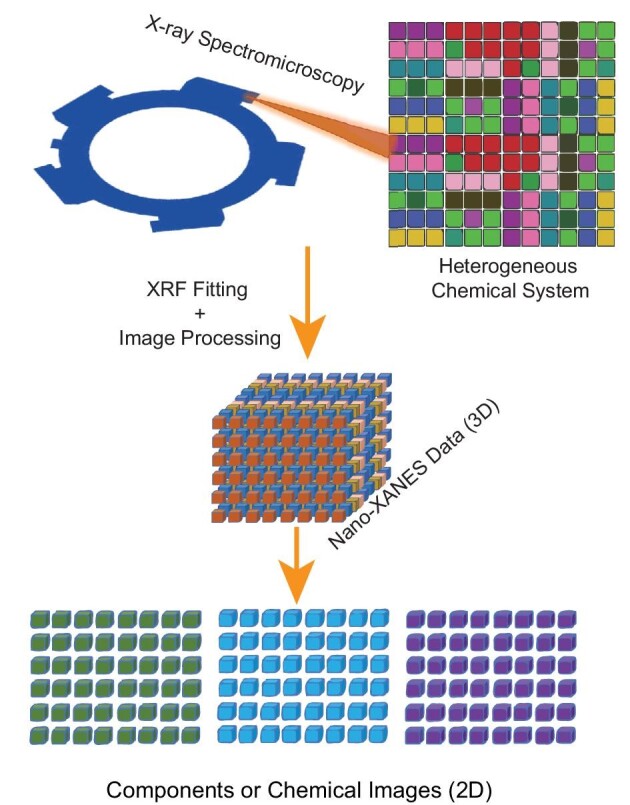
Analysis of spectromicroscopy data. A heterogeneous chemical system studied using the scanning probe spectromicroscopy technique produces multi-energy (50+) XRF maps. Three-dimensional nano-XANES data are created from the XRF maps by fitting and image processing. Further analysis of nano-XANES data results in chemical images and component maps showing the individual components in the sample.

Before we describe the specific details, it is valuable to give a high-level overview of the data structure and analysis workflow. A 2D raster scan is performed at regular (*X*, *Y*) grids or pixels for typical XRF imaging or nano-XRF imaging. A full XRF spectrum (or multiple spectra if a multi-element XRF detector is used) is collected at each pixel. This 3D dataset is reduced to 2D elemental images by determining the integrated XRF peak intensity. The integrated peak intensity for each element is determined through XRF peak fitting.^14^ The elemental images can be converted into 2D concentration images if the absolute quantification of the integrated intensity is achieved using XRF reference standards or calibrated samples. As schematically described in Fig. 1, the nano-XANES imaging performed XRF imaging at multiple energies across specific absorption energy. Thus, the nano-XANES imaging produces a 4D dataset at the (*E*_incident_, *X*, *Y*, *E*_fluorescence_) measurement grids. After performing the peak fitting, the 4D dataset is reduced to multiple 3D datasets, where the reduced 3D dataset contains the integrated XRF intensity over specific elements at (*E*_incident_, *X*, *Y*) grids. These 3D datasets are further reduced to a set of 2D chemical or speciation images by performing pixel-wise XANES analysis or matrix decomposition methods. To support complex visualization of the 4D dataset and perform a suite of computational analyses, we developed an integrated software package named XMIDAS (X-ray multimodal image data analysis software). Creating XMIDAS aims to minimize human inputs in multimodal data analysis, interlinking conventional analysis methods with machine learning algorithms, and provide a single platform to bridge the multimodal imaging and XANES analysis methods. The reproducibility and portability of the data analysis workflows are ensured by creating a graphical user interface and sharing the source code with the scientific community. In the remaining sections, we illustrate how these workflows are used for the speciation of heterogeneous chemical systems at the nanoscale.

## Materials and methods

### Sample preparation

Pyrite (Pyr) and lithium iron phosphate (LFP) were obtained from Sigma Aldrich (St. Louis, MO, USA). Hematite (Hem) nanoparticles and stainless steel (SS) nanoparticles were purchased from US Nano Research (Houston, TX, USA).

A heterogeneous nanoparticle aggregate was prepared by physically mixing the four different iron compounds (Pyr, Hem, SS, and LFP). The particles were first dispersed in acetone (5 mg in 1 mL) and mixed by ultrasonication (5 min). About 5 μL of the mixed dispersion were drop-casted onto a silicon nitride membrane window (Norcada, Edmonton, Canada). The solvent was dried off in the air, and the substrate was mounted onto a custom pin before loading into the microscope. The microscope chamber was backfilled with He gas at about 1/3 atm pressure. Particle aggregates suitable for spectromicroscopy studies were selected based on the size and data collection time. So the exact fraction of each starting material is unknown.

### Cell sample preparation

Fe(III) adsorbed to carbon nanoparticles (Fe/CNP) were synthesized by following the previously published method.^22^ The approximate surface coverage of Fe on CNP was 10 μmol/m^2^. Fe/CNP particles were suspended in ultrapure deionized H_2_O (MilliQ H_2_O) at a 200 μg/mL concentration and dispersed by ultrasonication for 2 min.

THP1 monocytes were maintained in RPMI1640 medium containing 10% FBS and 1% pen/strep at 4 × 10^5^ to 1 × 10^6^ cells/mL with 5% CO_2_ at 37°C. SiN windows were washed with HPLC-grade acetone and ethanol for 2 min each, then coated with 0.01% poly-lysine for 5 min at room temperature, and then sterilized under UV light for 30 min. SiN windows were placed at the bottom of the 96-well culture plate, then 150 μL  of cells per well (5 × 10^5^ cells/mL with 7.5 ng/mL phorbol 12-myristate 13-acetate) were added. Cells were cultured for 24 h and then treated with 10 μg/mL Fe/CNP nanoparticles for 6 hrs. SiN windows were carefully taken out, air dried, and stored at 4°C. The X-ray imaging of the biological samples in our study was performed within 10 days of the immobilization.

### XRF imaging

Scanning XRF imaging measurements were conducted at the Hard X-ray Nanoprobe (HXN) beamline at the National Synchrotron Light Source II (NSLS-II) at Brookhaven National Laboratory. A complete description of the HXN beamline and its end station instruments can be found elsewhere.^23^ HXN's standard nano-focusing configuration using a Fresnel zone plate (FZP) was utilized for XRF measurements. A monochromatic X-ray beam was pre-focused, in both horizontal and vertical directions, onto a secondary source aperture, which defines the effective source size for the nano-focusing. A focused X-ray beam (∼40 × 40 nm at 12 keV) was produced by the FZP with a 30 nm outermost zone width. XRF and the transmitted (DPC) data were simultaneously collected using a three-element silicon drift detector (Vortex, Hitachi, Inc.) and a pixel-array detector (Merlin, Quantum Detectors, Inc.), respectively. The XRF data were fitted using PyXRF.^14^ DPC analysis was carried out using in-house analysis programs^24^^,^^25^ and the resulting image data were exported to XMIDAS for further analysis.

### Nano-XANES imaging

#### Data acquisition

A detailed methodology for nano-XANES data collection was reported previously.^10^ XRF maps are collected across the Fe–K absorption edge at 7.112 keV (from 7.08 to 7.20 keV with 73 energy points). The total acquisition time for the model sample was about 24 h. The individual XRF maps were processed by fitting the XRF peaks and producing 3D image stacks, where each voxel contains the integrated Fe XRF signal. The individual Fe maps were spatially aligned by performing an image registration correction,^26^ which is necessary because the focused beam position with respect to the sample could be shifted while changing the incidence X-ray beam energy. Chemical or oxidation-state images are produced from the aligned XRF image stacks using component analysis and XANES fitting analysis.

#### Component analysis

The number of significant components in the nano-XANES data was estimated using the singular value decomposition (SVD).^27^ After identifying the number of significant components (*k*), the 3D stack is decomposed into individual 2D component maps using principal component analysis (PCA), NMF, or other decomposition methods available in the scikit-learn library.^28^ In particular, NMF^29^ is suitable for nano-XANES data because of the positivity constraint during the factorization. The component maps are then used to generate the component spectrum that resembles a XANES spectrum. For decomposition analysis, the 3D nano-XANES matrix ($e \times x \times y)$ was restructured to a 2D matrix V $(e \times p$) and factorized into two new matrices (*W* and *H*) using Equation 1 for NMF.

(1) \begin{equation*}
{\mathrm{\ }}{V}_{e \times p} = {W}_{e \times k\ }\ {H}_{k \times p\ }{\mathrm{\ }}k \ll e
\end{equation*}

The component spectra and images were computed from the *W* and *H* matrices. For example, the model sample stack with 73 energy points and 160 × 160 points was converted to a 73 × 25 600 matrix before factorization. The factorized matrix with five significant components then has a shape of a 5 × 160 × 160 matrix. In other words, a 73 × 160 × 160 matrix was reduced to a 5 × 160 × 160 matrix. The component spectra were normalized and compared with the reference library to generate a Pearson's correlation matrix.

#### Chemical imaging

Chemical maps are generated from the nano-XANES stack by fitting with a set of reference standards. After identifying the potential number of components from NMF, a combinatorial linear combination fitting of the nano-XANES spectrum was used to determine the chemical species. For an unknown sample, one can use a combinatorial fitting approach to identify the chemical phases in the sample.^30^ NMF analysis identified four distinct components in the sample, and we performed a combinatorial fitting of the nano-XANES data with a library of 11 reference standards (Supplementary Table S1, Supplementary Fig. S9). For higher computational speed, the 2D XANES data was binned (downsized) before the combinatorial fitting. After examining the *r*-factor, reduced χ^2^ statistics, and manual inspection of single-pixel fittings, maximum probable candidates were selected for chemical imaging. Chemical images are generated from the nonnegative least-squares fitting coefficients of the reference standards in 2D that represent the abundance of that component in the sample. All the image and spectrum calculations are performed using the in-house software package, XMIDAS. The source code is available on the GitHub repository (https://github.com/NSLS-II/xmidas), and the installation instructions are found at https://nsls-ii.github.io/xmidas/installation.html. An overview and structure of the interface are shown in Supplementary Figs. S1–S4.

## Results

### XRF analysis and elemental correlations

**Fig. 2:**
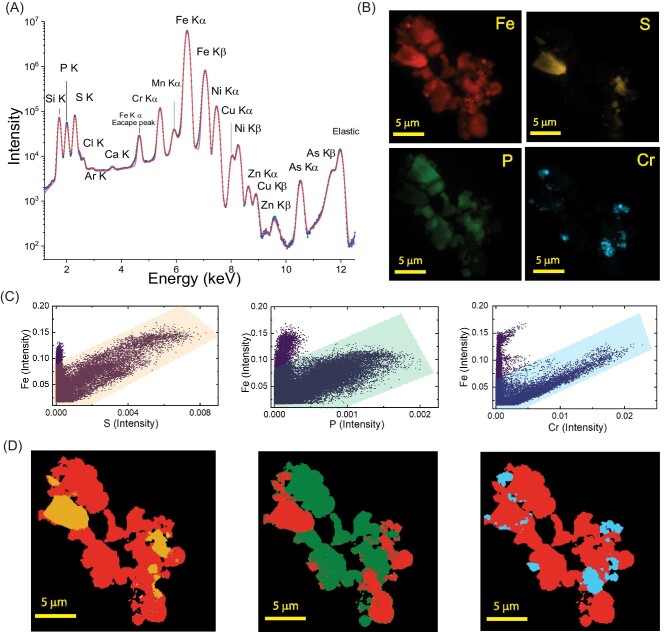
Nano-XRF analysis of the model particle aggregate. A. Average XRF spectrum of the particle aggregate and fit (in red). B. XRF maps show the abundance of major elements in the sample. C. Correlation of XRF intensities of Fe–S, Fe–P, and Fe–Cr at each point. The shaded regions are identified as points with a linear relationship between the elements. D. The shaded points in C were back-projected to 2D XRF to determine the spatial coordinates of specific Fe–S, Fe–P, and Fe–Cr correlations.

XRF imaging analysis and elemental correlations are illustrated using an artificially created heterogeneous aggregate consisting of Pyr, LFP, Hem, and SS nanoparticles. The goal of XRF mapping was to identify the abundance of each element in the aggregate and create 2D correlation maps. The summed XRF spectrum is fitted using PyXRF, accounting for XRF emission lines, elastic scattering, Compton scattering, and instrumental artifacts such as pile-up and escape peaks (Fig. 2A). Apart from the elemental emission lines, an emission line at 4.66 keV was identified as an escape peak from the silicon drift detector caused by Fe–K alpha emission. Conveniently, PyXRF detects the escape peak artifacts, knowing the detector element. Similarly, any pile-up peaks may be parameterized in the fit to avoid spectral contamination into elemental lines, although we did not observe noticeable pile-up peaks in this measurement. After all the elements and artifact peaks are assigned, PyXRF performs least-square fitting for pixel-wise peak fitting only for the peak intensities, resulting in a much faster fitting speed than nonlinear fitting.

The major elements present in this sample were Fe, S, P, and Cr, and their XRF maps are shown in Fig. 2B. These elemental maps suggest that the aggregate has at least three starting materials because the presence of Hem (Fe_2_O_3_) cannot be confirmed with XRF. We also noted a trace abundance of elements (Cl, Ca, Zn, Cu, and As) in the spectra due to the impurities mainly in Pyr and other starting materials noted by the manufacturer (Supplementary Fig. S5). The correlation between fluorescence intensities of elements is used to evaluate their spatial relationship against stoichiometry. Because all the constituents contain Fe, regions outside the Fe map are considered as background for elemental correlation analysis. First, a scatter plot is generated to show the relationship between XRF intensities between two elements at each pixel (Fig. 2C). Next, the points with a visual relationship were interactively selected (shaded regions in Fig. 2C) and back-projected to the 2D XRF map coordinates, resulting in a binary mask (Fig. 2D). For example, a Fe/S correlation map in Fig. 2C suggests a linear relationship between Fe and S intensities in the shaded region (in yellow), and other points showed no apparent relationship. The corresponding points in 2D clearly showed particles with a specific relationship (in yellow), and the regions outside the shaded area are shown in red. A similar analysis was performed to identify regions with Fe/P and Fe/Cr correlation. The elemental sensitivity of XRF maps is helpful in predicting chemical phases, but element correlations alone are not sufficient to identify chemical speciation. For example, the Fe/S correlation implies numerous possibilities, such as Fe sulfides, sulfates, thiolates, etc. Therefore, chemical state-sensitive nano-XANES measurement was performed by multi-energy XRF mapping.

### Nano-XANES

This section illustrates nano-XANES data analysis workflow development using the model sample. In practice, nano-XANES is 4D data generated by collecting 3D nano-XRF at several energy points across the absorption edge of the element of interest. A 3D grid array of multi-energy XRF maps (also called an energy stack) is created by XRF peak fitting, followed by image processing and alignment. The following sections describe methodologies to reduce nano-XANES data to chemical state maps.

### Component analysis

**Fig. 3:**
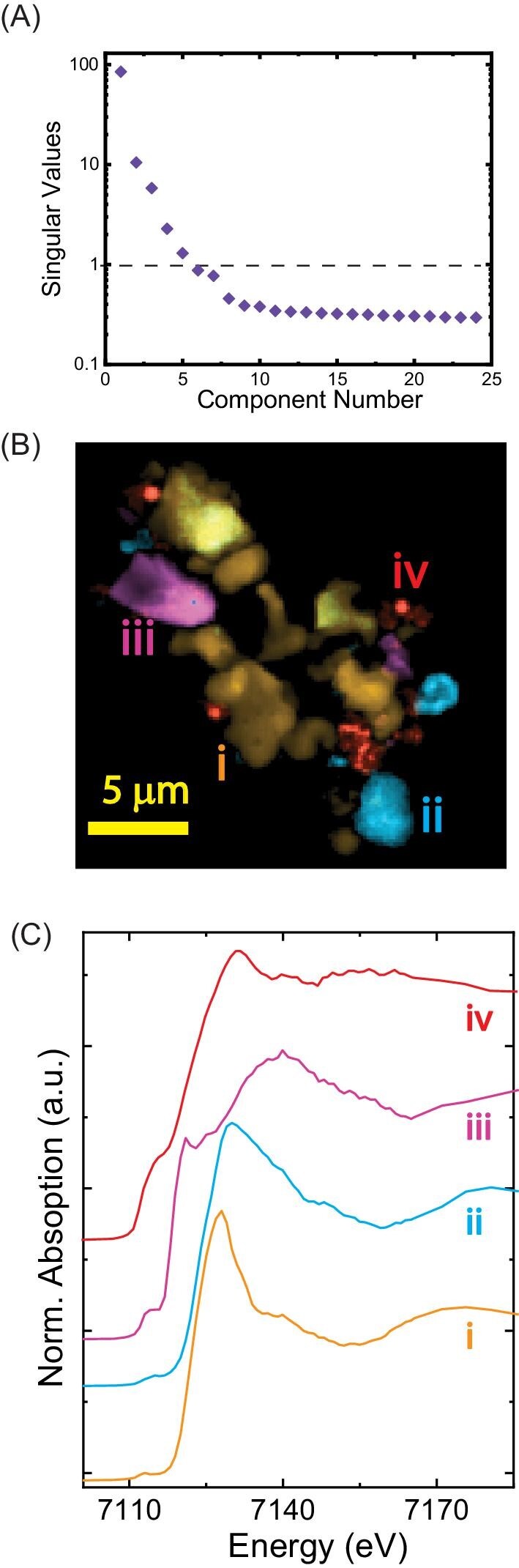
Component analysis of nano-XANES data. A. Scree plot of singular values to determine the number of significant components in the data. B. Composite map showing decomposition maps using NMF (nonnegative matrix factorization) method. C. Decomposed Fe–K edge XANES spectra from NMF analysis corresponding to the regions in B.

The goal of using machine learning algorithms to analyse nano-XANES is to differentiate the regions with identical spectral signatures. Here we tested and optimized several decomposition algorithms to decompose the 3D nano-XANES data into a small set of 2D matrices. An important estimator for the component analysis is the minimum number of 2D matrices (components) that maximally represent the data. The number of significant components in the nano-XANES data was estimated by the SVD^31^ method. The singular value of each component is plotted in Fig. 3A (called a scree plot because of the shape). By examining the scree plot, we determined that the first five components explained >90% of the data here and were used as an optimizer for decomposition analysis. In addition, component mapping using more than five components was manually inspected and ruled out as noise.

The composite map of components two to five is shown in Fig. 3B, and the first component is omitted because, in theory, it is the mean of all the components. The component spectrum from each mapped region showed clear differences in edge maximum and shape, suggesting different chemical phases. The component spectra may be compared with the reference spectrum library to suggest potential chemical phases in the decomposition map. For qualitative analysis, a correlation matrix of the component spectrum (normalized) against the reference library (Supplementary Table S1) was created employing Pearson's correlation coefficient (Supplementary Fig. S2). The matrix suggests that the first component has a high correlation to LFP, and the third component has a high correlation to Pyr. The correlation of the other two components with the reference spectra was more ambiguous. The second component spectrum showed similarity to a few Fe(III) standards. Likewise, the fourt component spectrum showed a match with Fe-phosphide, Fe(II), and Fe(0) standards. The deconvoluted component spectra also showed a high correlation to each other (Supplementary Fig. S6B). It is worth mentioning that an experimentalist may also figure out the similarities in spectral shapes. Still, our goal is to establish the workflow with minimal human inputs for larger, complicated datasets**.**

### Chemical mapping

Chemical mapping aims to identify chemical phases at each pixel by fitting the spectrum against known reference spectra. The linear combination fitting of the spectrum is the benchmark method for XANES analysis. However, identifying the probable reference standards for the fitting is very crucial in computing “realistic” chemical state maps. If one has prior knowledge about the sample (e.g. phase identification from X-ray or electron diffraction), the number and type of reference standards may be selected accordingly. Otherwise, combinatorial fitting with a large pool of reference standards can be used to find the solution by monitoring the goodness of the fits by statistical measures, although this approach does not directly assess the fit accuracy and may not produce a unique result.^30^ Therefore, we used the information from the NMF and XRF results to select the group of possible reference spectra from the library, which contained 11 individual standards (Supplementary Table S1). The goal of the combinatorial nano-XANES fitting methodology here was to optimize the parameters using the model sample. We performed an automated combinatorial fitting (>1000 combinations) of the nano-XANES data. Because the potential chemical phases are known to us, the reliability and accuracy of the methodology can be assessed and optimized. The results of the combinatorial fitting are ranked based on reduced χ^2^, as shown in Table 1. The solution to the fit ranked second on the list. The unexpected component in all the other solutions was a Fe(III) compound with ≤5% abundance. Therefore, the existence of this phase was manually inspected using 2D chemical maps and concluded as noise. These results suggest the need for further optimization of the combinatorial method for complete automation.

**Table 1.** Combinatorial XANES fitting results (SS: stainless steel; Hem: hematite; Pyr: pyrite)

Name	Set of reference standards	The normalized coefficient for each component	*r*-factor	reduced χ^2^
(i)	(Goethite, LiFePO_4_, SS, Pyr, Hem)	(0.05, 0.50, 0.18, 0.11, 0.16)	0.000199	0.00253
(ii)	(LiFePO_4_, SS, Pyr, Hem)	(0.50, 0.18, 0.12, 0.20)	0.000174	0.00256
(iii)	[Fe_2_(SO_4_)_3_, LiFePO_4_, SS, Pyr, Hem]	(0.02, 0.50, 0.17, 0.12, 0.18)	0.000173	0.00257
(iv)	(LiFePO_4_, FePO_4_, SS, Pyr, Hem)	(0.50, 0.01, 0.18, 0.12, 0.18)	0.000173	0.00258
(v)	(Maghemite, LiFePO_4_, SS, Pyr, Hem)	(0.04, 0.50, 0.18, 0.12, 0.16)	0.000175	0.00259

**Fig. 4:**
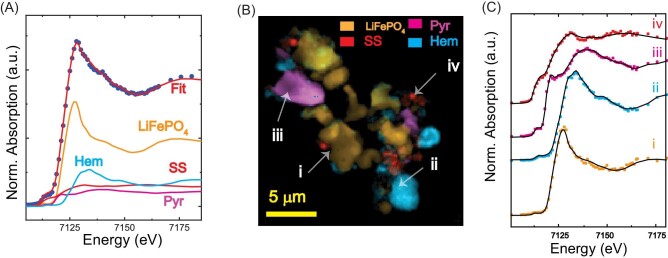
Chemical imaging using spectromicroscopy A. The average nano-XANES spectrum and linear combination fit. Each reference standard spectrum is scaled according to its weighted contribution to the fit. B. Composite chemical image representing the abundance of each chemical phase. C. Single-pixel (50 × 50 nm^2^) spectrum and fit from each region (LFP—LiFePO4, SS—stainless steel, Pyr—pyrite, Hem—hematite).

The summed XANES spectrum and the fits are shown in Fig. 4A, which displays the goodness of the fit and weights used for each reference spectrum (Fig. 4A). Next, the chemical state maps were generated by fitting the XANES spectrum at each pixel. Chemical maps are the fitting coefficients of the reference compound in 2D that show its spatial distribution (Fig. 4B). The major phase in the particle mixture is LFP (∼50% spectral fraction) aggregated with the other phases, followed by 20% Hem, 12% Pyr, and 18% SS nanoparticles (spectral fractions). These results were reproduced in ATHEANA program by fitting the integrated XANES spectrum (Supplementary Fig. S10). The reliability of the chemical mapping is related to the quality of the single-pixel spectrum and the fit. Thus single-pixel spectrum and fit from each region are presented in Fig. 4C. Note that the model sample is rich in Fe, and a quantifiable single-pixel spectrum may be acquired with a low acquisition time (50 ms/point here). But for trace metal analysis, one may integrate the pixels (binning) or use higher dwell times for statistically reliable quantification by improving the signal-to-noise ratio. We tested the possibility of obtaining chemical state maps from poor S/N data was tested by adding random Gaussian noise to the existing data (Supplementary Fig. S11). Chemical state analysis was performed using two datasets with standard deviation 3× and 10× larger than the original data. The resulting chemical state maps that fitted with the reference standards were identical to the original data with noticeable noise (Supplementary Fig. S11). However, the NMF analysis could not resolve the components from the noisy data. The results test suggests the need for a high-quality single pixel spectrum for component analysis and chemical mapping feasible by fitting with reference standards.

To reduce acquisition time and dose, several beamlines collect multi-energy point XRF maps (∼10 energy points) for oxidation state mapping, instead of complete nano-XANES (>50 energy point). To prove the feasibility of using XMIDAS for multi-energy point data analysis, we generated 12 energy point data from the original data (73 points). The energy points are selected based on the characteristic peaks in the Fe spectrum library. The NMF analysis with mutli-energy point data showed comparable results original data (Supplementary Fig. S12) as all the components were deconvoluted. Similarly, chemical map analysis and quantification results were reproduced using the multi-energy point data (Supplementary Fig. S13 and Supplementary Table S2). Hence, the XMIDAS workflow is extendable to micro/nanoprobe beamlines for multi-energy point mapping experiments.

### Spectromicroscopy to characterize nanoparticles in biological cells

**Fig. 5:**
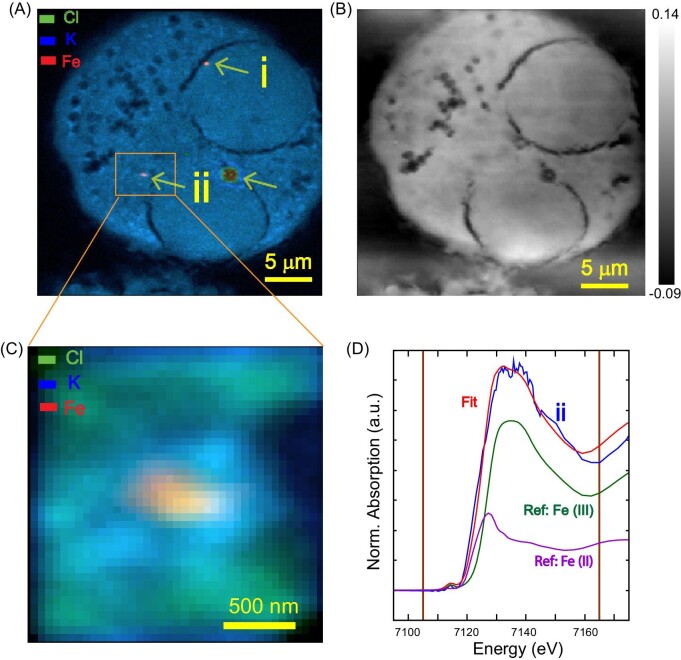
Interaction of simulated air pollution particles [Fe(III) on carbon nanoparticles, Fe/CNP] with lung macrophages. A. Composite XRF map of Cl, K, and arrows highlight Fe/CNP nanoparticles (120 × 120 nm^2^ step size). B. Differential phase-contrast (DPC) image of the cell. C. High-resolution image (50 × 50 nm^2^ step size) of a Fe/CNP particle in association with the cell. D. Linear combination fit of XANES spectra from Fe/CNP particle with Fe(III) and Fe(II) reference standards. Data points inside the vertical lines were used for fitting.

This experiment aimed to show that X-ray spectromicroscopy tools can be used to examine the migration and fate of nanoparticles after exposure to biological cells. After optimizing the data analysis workflow to examine heterogeneous chemical species, we applied this technique to locate and characterize trace nanoparticles associated with a biological cell. Here we studied the interaction of carbon nanoparticles with surface-adsorbed Fe(III) (Fe/CNP), which represents a synthetic component of nano-sized airborne particulate matter, with alveolar macrophages.^22^^,^^32^ Biologically abundant elements such as P, S, K, Cl, etc., were mapped to determine the overall morphology of the cells, and metal-bearing nanoparticles were mapped in the hard X-ray energy range. In Fig. 5A, Cl and K maps show the cell morphology and organelles, whereas the Fe/CNP nanoparticles^32^ are also visible in the Fe map. In addition, the transmitted X-ray intensity was used to create a DPC image (Fig. 5B), which is in agreement with the fluorescence maps. Because DPC maps are sensitive to the change in the refractive index of the medium, other cell components may be visualized, independent of fluorescence imaging. The DPC images can be used to interpret the organelles and the 2D location of the nanoparticles.

The Fe-bearing nanoparticles in/on the cells were further analysed using nano-XANES. The integrated XANES spectra from two particles are shown in Fig. 5D in comparison with Fe(III) and Fe(II) reference standards (Fe/CNP and LiFePO_4_, respectively). The particles in contact with the cells showed a clear difference compared to the particle spectra collected before cell exposure [Fe(III) ref.] that indicated only Fe(III). The linear combination fit showed a 29% Fe(II) contribution, suggesting the possibility of Fe reduction associated with cell exposure. Note that the fit was performed with reference compounds to quantify Fe oxidation states. Expansion of the reference standard library with Fe species and phases expected in biological systems is required for chemical phase analysis. This experiment demonstrated that nanoscale trace particles could be located and examined in heterogeneous biological environments using multi-model X-ray nanospectroscopy.

## Discussion

### Multimodal nano-XRF and nano-XANES

We illustrated that a combined analysis of nano-XRF and nano-XANES provides a detailed understanding of the speciation of the heterogeneous chemical systems. Having both the XANES spectrum and XRF spectrum at the same point serves as a validation tool for chemical speciation from nano-XANES. As mentioned earlier, the accuracy of chemical images from nano-XANES relies on selecting adequate reference standards. But having XRF maps of the same sample facilitates the selection process to create a reasonable XANES reference spectrum library. In our example, there were regions with strong Fe–P correlations. Thus three available Fe–P compounds [LFP, Fe(III)-phosphate, and Fe-phosphide] were included in the library. Similarly, Fe–S and Fe–Cr compounds were included in the fit. On the other hand, some reference compounds such as Fe-silicates and aluminosilicates were excluded from the fit. However, Fe-oxides, carbides, nitrides, organic-Fe, etc., cannot be excluded from the fit because low-*Z* elements (*Z* < 14) maps are not available. For instance, there were regions where only Fe was detected in the model sample, which may be interpreted as either Fe(0) or Fe + low-*Z* element composition (Supplementary Fig. S8). As a result, available Fe-oxides, Fe–C, and metallic Fe compounds were included in the reference library. Thus, nano-XRF maps can add reasonable constraints to XANES fitting and, ultimately, the nano-XANES analysis to precisely identify the component. Finally, the XRF spectrum may confirm the XANES speciation in the same region. For example, if the XANES spectrum fit results suggest a Fe–S compound, the presence of S in the XRF spectrum in the same area can be validated. The masking tools and interactive visualizations in our software package enable such validations, and in the future, this process will be automated. It should be noted that the generation of chemical maps is dependent on the availability of reference standards that requires either a large pool of reference spectra or prior knowledge of the unknown components. In the future, the use of computational modeling and data sharing depositories could be implemented in the workflow to improve this process.^33^ A practical challenge is worth mentioning here is the acquisition time for nano-XANES. As the experiment is equivalent to collecting 60–70 XRF maps at different energy points, the total acquisition time is >5 h, depending on the sample strength.

### Component analysis and conventional nano-XANES analysis

One limitation of the conventional nano-XANES analysis using linear combination fitting is the need for *a priori* sample knowledge to select appropriate reference standards for chemical imaging. When the sample is unknown, the availability and selection of a potential reference spectrum library is a cumbersome task. To address this challenge, we showed that unsupervised dimensionality reduction algorithms could aid spectromicroscopy data analysis by spectrum-based decomposition (or clustering). PCA analysis is a common method used to decompose spectromicroscopy data in the past.^16^^,^^18^ Mirna *et al.* illustrated the application of PCA to examine soft X-ray spectromicroscopy data (STXM).^18^ NMF and independent component analysis (ICA) was also used in prior studies for micro-XRF and STXM data as well.^16^^,^^18^ The decomposed eigenvectors and eigenvalues in PCA have no direct relationship to the XANES spectrum due to the negative values. Alternatively, the NMF method with positivity constraint outputs the eigenvectors and eigenvalues that can easily be transformed into interpretable XANES spectra. This methodology directly compares the component spectra to XANES reference spectra. In addition to PCA, NMF, and ICA, we implemented and optimized a few other decomposition methods such as factor analysis,^34^ truncated-SVD,^31^ and dictionary learning^35^ to the toolkit. We also confirmed that the PCA results obtained from XMIDAS and the Mantis spectromicroscopy program are identical (Supplementary Fig. S7). The nano-XANES from the model sample are high-quality single-pixel spectra; therefore, the decomposition models’ results were identical. So further optimizations are warranted for noisy datasets in the future.

Although the decomposition models yield decomposition of the spectrum and image data, the prevalent use of these methods is not widespread in the spectromicroscopy literature. Therefore, we illustrated how this information could support the chemical speciation of similar heterogeneous metallic systems. A direct comparison of chemical state maps and NMF component maps (Fig. 3B) clearly agree with the speciation using conventional modeling (Fig. 4B). Because the NMF analysis does not require knowledge of sample composition, the NMF model is valuable for decision making during the experiment and narrowing down possible reference standards during the post-analysis. Thus setting this benchmark with the model sample with known speciation is essential in the tool development. The NMF modeling also addresses some caveats of XRF correlation analysis. Here XRF correlation analysis showed three elemental correlations (Fe–S, Fe–Cr, and Fe–P) in the aggregate due to the lack of low-*Z* elemental maps. But the NMF model based on XANES spectral signatures correctly predicted at least four chemical species in the aggregate. This analysis can be instrumental when the sample comprises two or more oxides, pure metals, or low-*Z* elements. In the future, sophisticated algorithms or artificial intelligence tools^36^ may be developed to recognize the decomposed spectrum using reference library input and XRF spectrum for semi-automated chemical speciation.

### Multimodal analysis of bio–nanoparticle interactions

Phase-contrast imaging was illustrated as another spectromicroscopy modality combined with XRF to study soft material interfaces with metallic systems. At the hard X-ray range, XRF mapping cannot image the morphology and abundance of soft materials such as biomolecules and polymers. However, the transmitted X-ray probe information simultaneously yields quantitative DPC images.^13^ This technique reveals the morphology of the whole sample, regardless of the constituent elements. Combining these modalities (nano-XRF, and DPC) is an avenue to study cell–nanoparticle interactions. Furthermore, speciation of the nanoparticles with nano-XANES data from the nanoparticles sheds light on the mechanism of any chemical interaction of nanoparticles in the cells. Here we identified spectral differences between pre-and post-cell exposure nanoparticles that indicated changes in Fe oxidation state, suggesting that the nanoparticles had some chemical interaction(s) with the cells. In contrast to the electron microscopy methods that typically require sectioning, conductive coating, and labeling, the established methodology allows direct interface measurement. The challenge in studying the interaction of nanoparticles with biological systems with X-ray nanoimaging techniques is the problems associated with penetration depth and sensitivity.^37^ Herein, we demonstrated that high sensitivity nano-XRF maps that can probe up to 10–30 μm are ideal for studying trace elements interfaced with cells. In comparison with soft-X-ray spectromicroscopy, where a few hundred nanometer-thin samples are required, a few micron-thick sections may be analysed using hard X-ray spectromicroscopy. However, self-absorption due to thick samples can result in incorrect quantifications and speciation, although it is not a significant factor in cell imaging. We are currently working on extending the self-absorption correction approach developed for 3D-tomography to the 2D XRF/XANES imaging.^38^ Although the 2D location of the nanoparticles associated with the cells is clear from this study, future tomography experiments are warranted to find the 3D location of the particles and to formulate the mechanism of intake and potential reactions.^39^ Radiation damage is a challenging problem in biological imaging with X-rays, although we did not observe any visible damage to the cells in our experiments. In comparison, hard X-rays are less damaging to cells than soft X-rays due to the high absorption cross-section of C, N, and O.^40^ Nonetheless, a detailed investigation in this area may require for nano-XANES in combination with 3D tomography (>24 h of exposure).

## Conclusions

We have demonstrated the technical capability and data analysis methodologies of multimodal spectromicroscopy in characterizing heterogeneous chemical mixtures. The technical challenges in spectromicroscopy data deduction (4D+ to 2D) were addressed by creating custom data analysis methodologies and the open-source toolkit. Our approach was to define and optimize the workflows with a heterogeneous model system with known chemical composition to benchmark the feasibility and accuracy. Visualizing multidimensional data is another challenge in spectromicroscopy, where both image and spectrum axes provide meaningful information about chemical speciation. The new graphical user interface solves this problem through interactive plotting, multi-correlation plots, and multicolor image overlays. The second thrust of this paper was to combine the conventional methods of spectromicroscopy analysis with well-established machine learning models for dimensionality reduction and clustering. We optimized several decomposition algorithms for spectromicroscopy data and their connection to the conventional analysis methods. In the future, advanced machine learning and artificial intelligence tools may be incorporated into the workflow for (semi) automated data analysis. Our ongoing developments include (i) selection of XANES reference standards using XRF elemental information, (ii) minimization of energy points for reliable chemical mapping, and (iii) better reference standard ranking system for combinatorial fitting. Finally, the application of the techniques to study bio–nano interactions was presented, where the sensitivity to both metallic nanoparticles and soft materials was utilized. The current study paves the way for future investigations on the distribution, transformation, and fate of nano/microparticles in biological systems using multimodal spectromicroscopy tools.^41^ We foresee the applications of this methodology in the fields of nanotoxicity, bio–nano imaging, and biomedicine.^42^^,^^43^

## Supplementary Material

mfac078_Supplemental_File

## Data Availability

The data underlying this article will be shared on reasonable request to the corresponding author.
