# Supplementary material for: Multimodal X-ray nano-spectromicroscopy analysis of chemically heterogeneous systems
Source: Metallomics. 2022 Oct 8;14(10):mfac078. doi: 10.1093/mtomcs/mfac078 (PMC9584160; doi:10.1093/mtomcs/mfac078)
Supplement: mfac078_Supplemental_File [file mfac078_supplemental_file.docx]

**Supporting Information**

Multimodal X-Ray Nano-Spectromicroscopy Analysis of Chemically Heterogeneous Systems

*Ajith Pattammattel^1*^, Ryan Tappero^1^, Dmitri Gavrilov^1^, Hongqiao Zhang^2^, Paul Aronstein^3^, Henry Jay Forman^2^, Peggy A. O’Day^3,4^, Hanfei Yan^1^, Yong S. Chu^1*^*


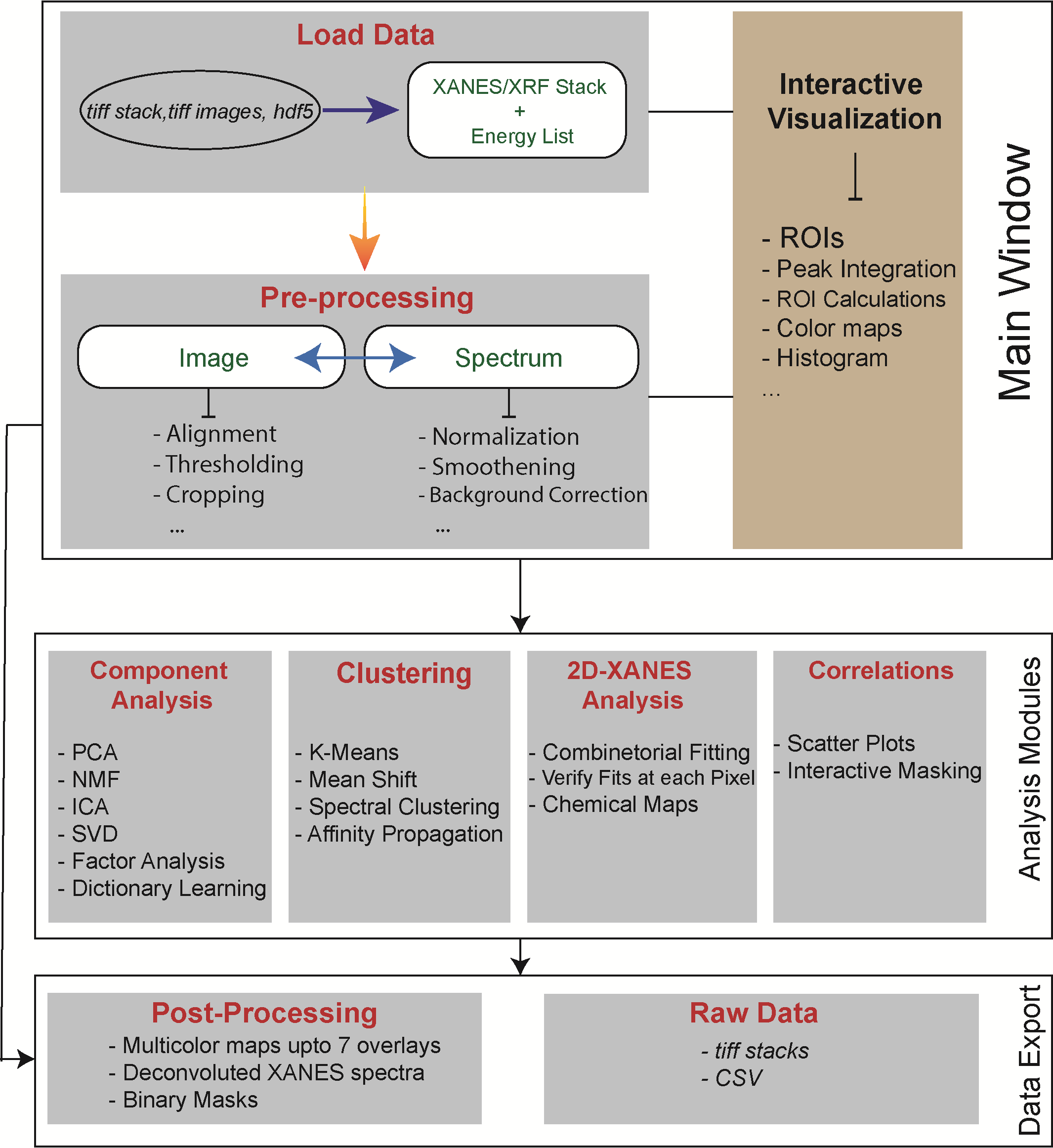


Figure S1. Structure of XMIDAS Program.


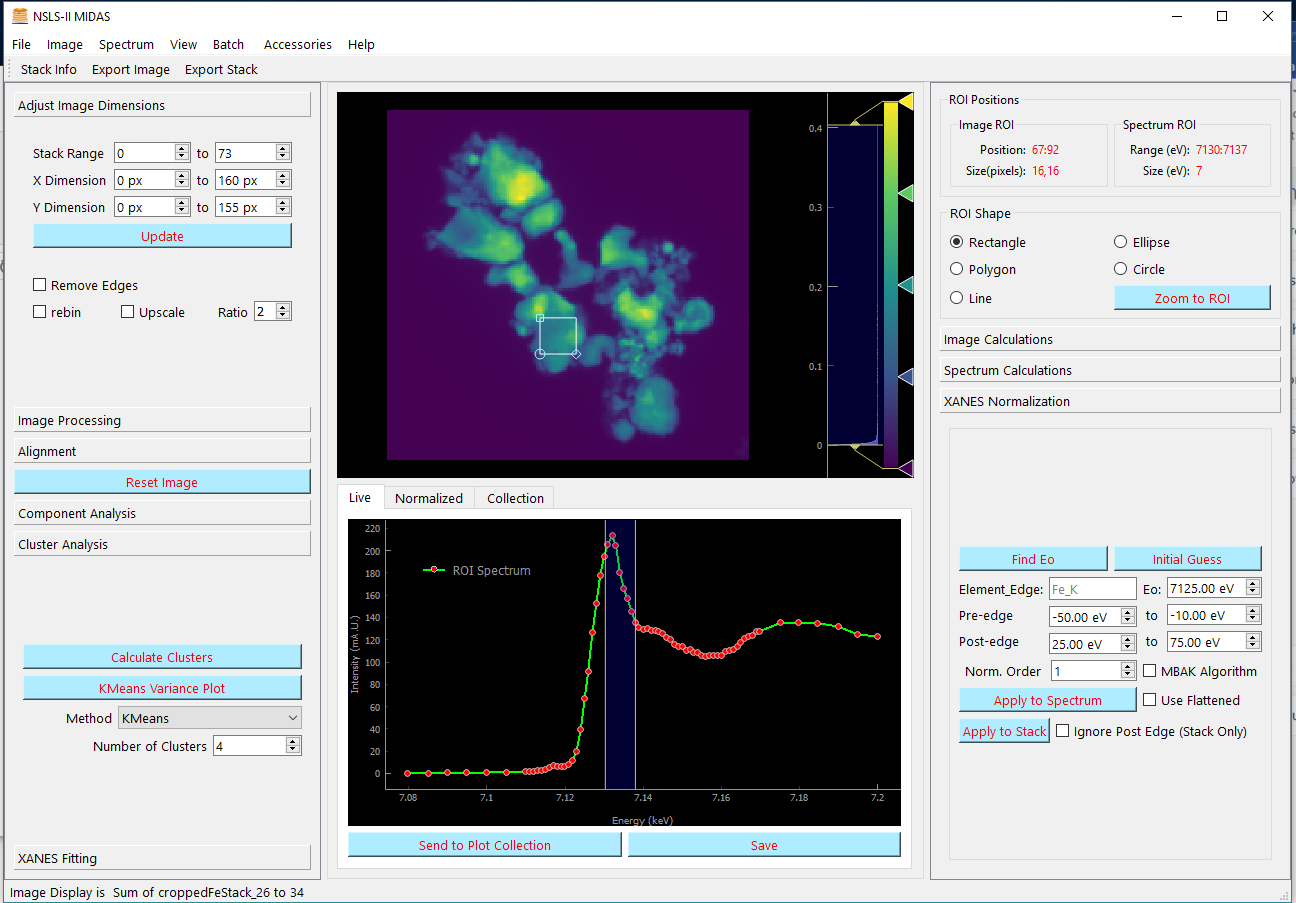


Figure S2. Snapshot of XMIDAS user interface.


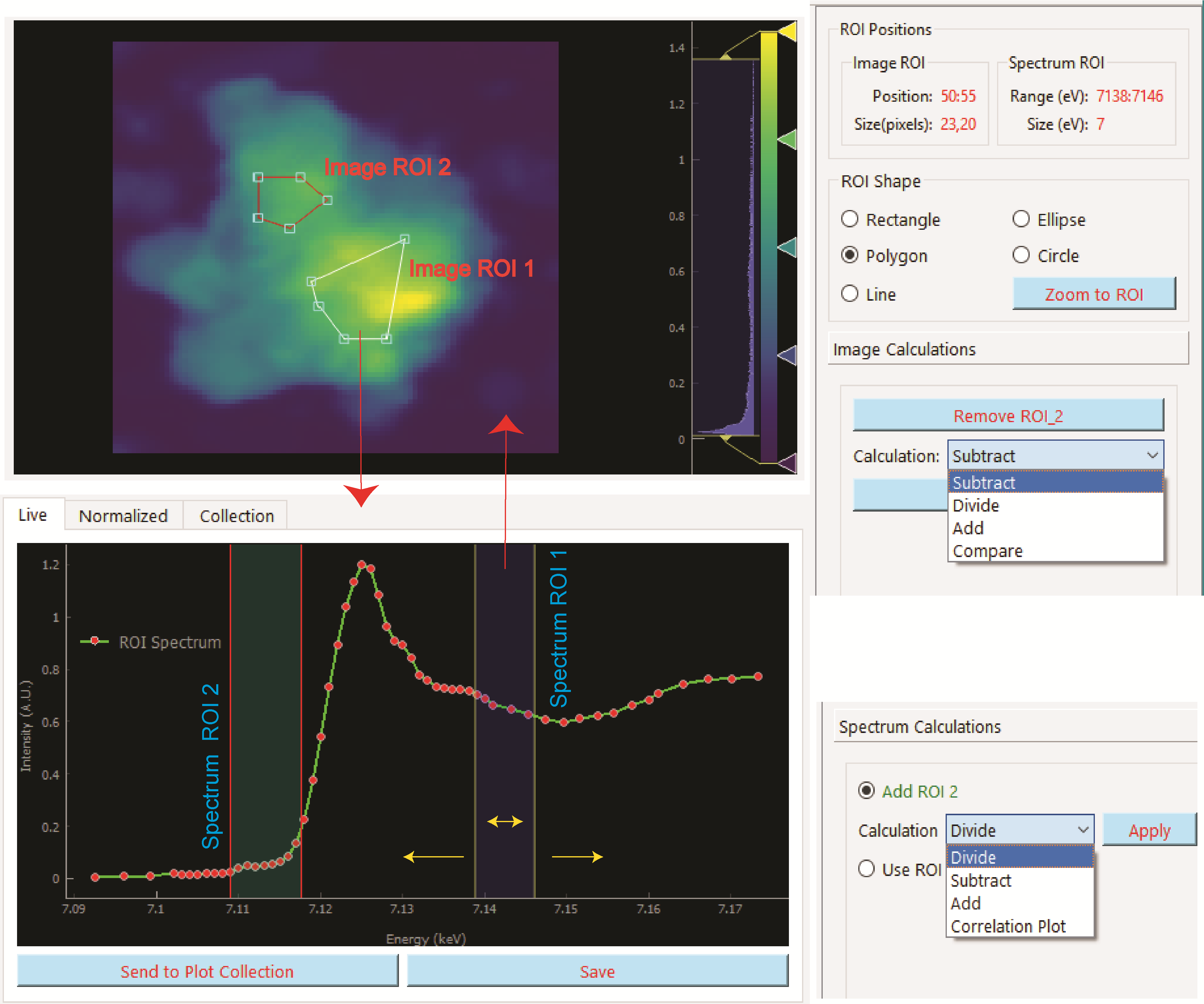


Figure S3. Interactive ROI tools on the image and spectrum panel are used for multi-dimensional visualization. The resizable, reshapable and translatable ROIs help the users to visualize the spectrum and image data at every point, in all dimensions.

A.
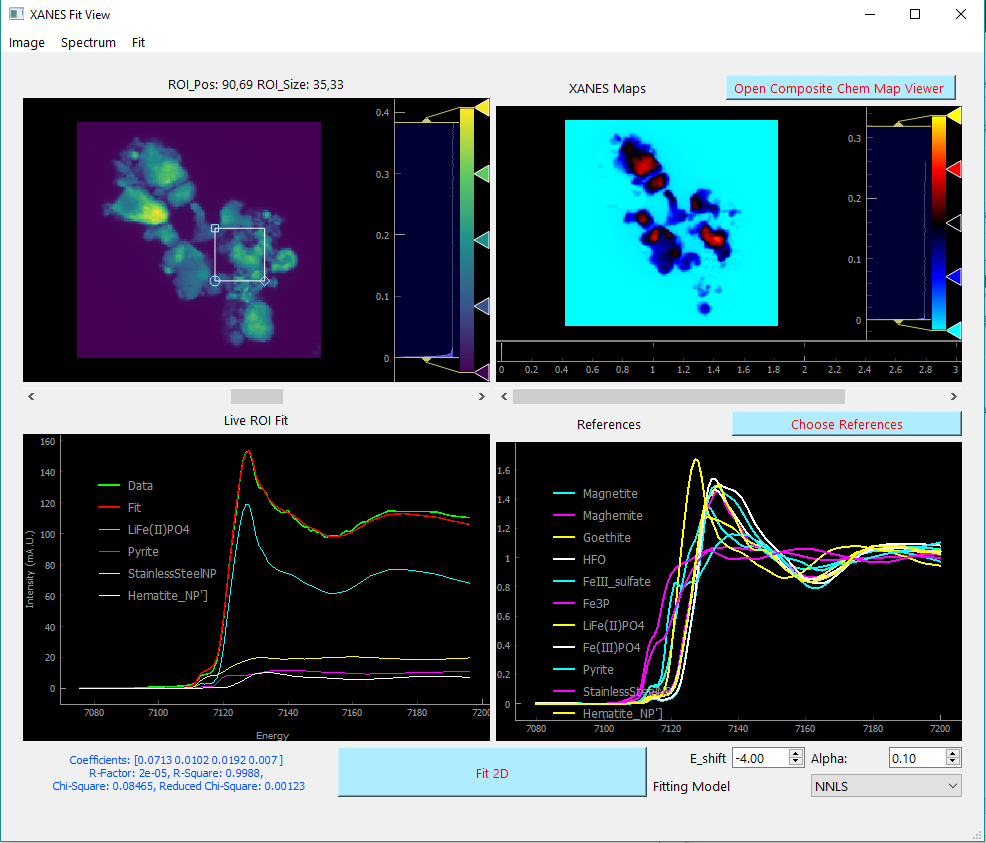


B.
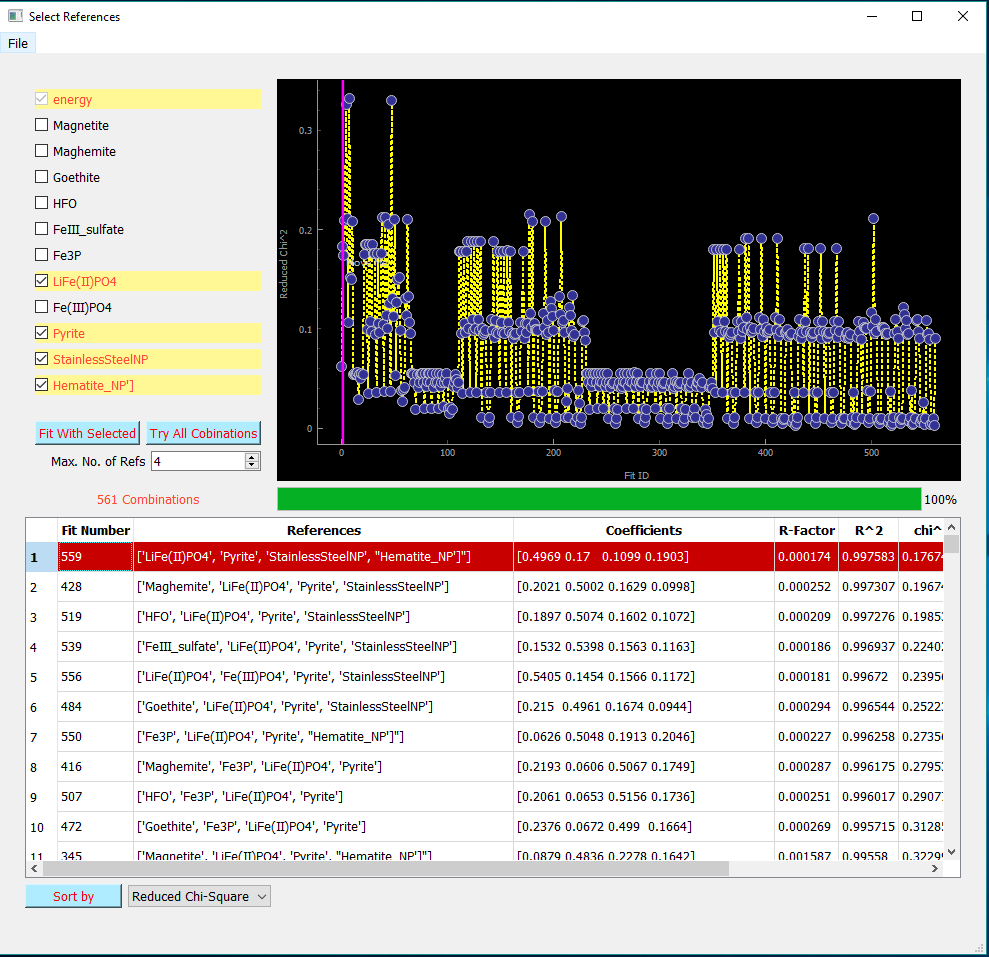


Figure S4. Nano-XANES fitting interfaces in XMIDAS. A. Live fitting and chemical state maps B. Interactively choose reference standards from the spectrum library.


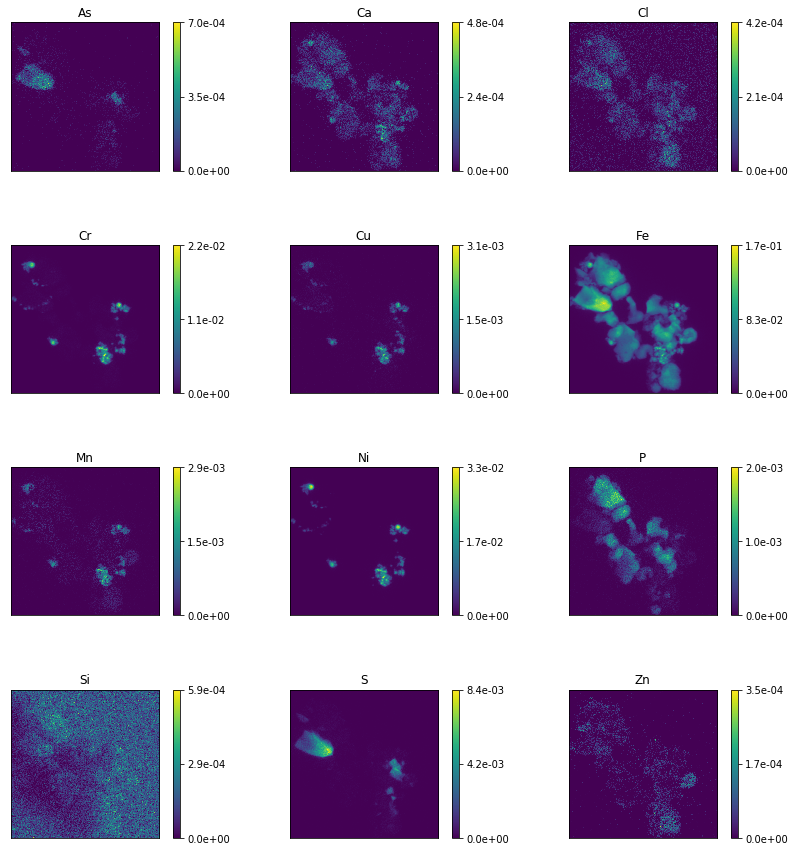


Figure S5. XRF map of all elements present in the sample. The color bar represents normalized fluorescence intensity, and the images are 22 μm x 22 μm in size.

A.
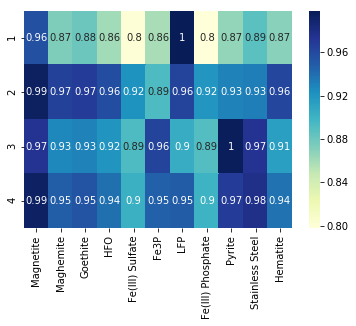


B.


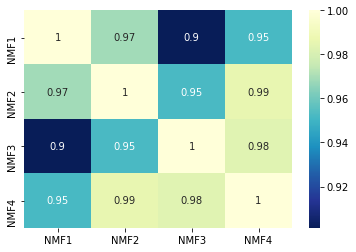


Figure S6A. Pearson’s cross-correlation matrix compares NMF component spectra with reference spectra to predict the chemical phases. B. Correlation between the NMF component spectra shows a high correlation to each other. (HFO- Ferrihydrite, LFP-Lithium Iron Phosphate)

A.
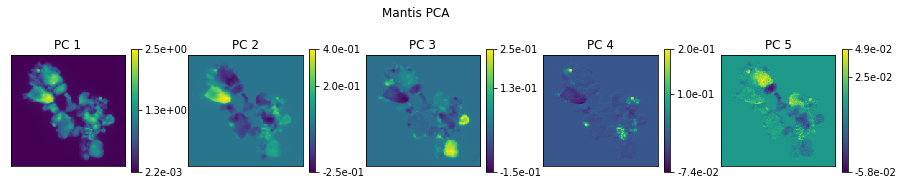


B.
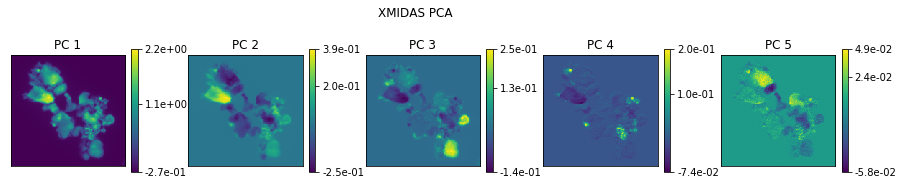


Figure S7. Comparison of PCA results from Mantis (A) and XMIDAS (B) programs show identical results


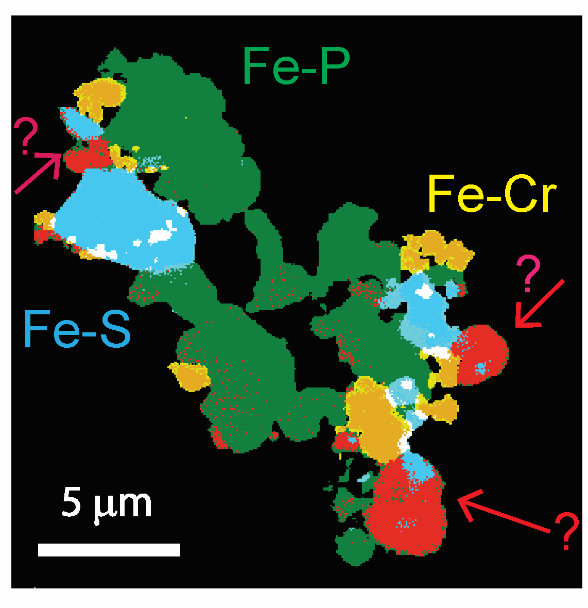


Figure S8. Overlay of regions with Fe-P, Fe-S, Fe-Cr correlation with Fe alone. The regions shown in red are not associated with any element detected in XRF.

**Table S1.** Reference spectra library used for combinatorial fitting of 2D XANES Data

| Compound | Description | Source | Data Collection Mode ^a^ | Ref. |
| --- | --- | --- | --- | --- |
| Stainless Steel | Fe(0) stainless steel nanoparticles (< 100 nm) | commercial  (US Research Nanomaterials, Inc.) | trans | This work |
| Magnetite | Fe^II^O,Fe^III^_2_O_3_ | natural | trans | ^1^ |
| Goethite | α-Fe^III^OOH | synthetic | trans | ^1^ |
| Hematite | α-Fe^III^O_2_O_3_ nanoparticles | (US Research Nanomaterials, Inc.) | trans | This work |
| Maghemite | γ -Fe^III^O_2_O_3_ | natural | trans | ^1^ |
| Ferrihydrite | amorphous Fe^III^(OH)_3_ | synthetic | fluor | ^2^ |
| Fe(III) / carbon NP | Fe(III) / carbon nanoparticles  (3 µmol Fe / m^2^ solid) | synthetic | fluor | ^2^ |
| Ferric Phosphate | Iron(III) phosphate tetrahydrate (FePO_4_ .4H_2_O) | commercial (Sigma Aldrich) | fluor | This work |
| Lithium Iron (II) Phosphate | LiFePO_4_ | commercial (Sigma Aldrich) | fluor | This work |
| Ferric sulfate | Fe_2_(SO_4_)_3_.5H_2_O | commercial (Sigma Aldrich) | fluor | ^2^ |
| Pyrite | FeS_2_ | commercial (Sigma Aldrich) | fluor | This work |
| Iron Phosphide | Fe_3_P | commercial (Sigma Aldrich) | fluor | This work |

*^a^* trans: transmission; fluor: fluorescence


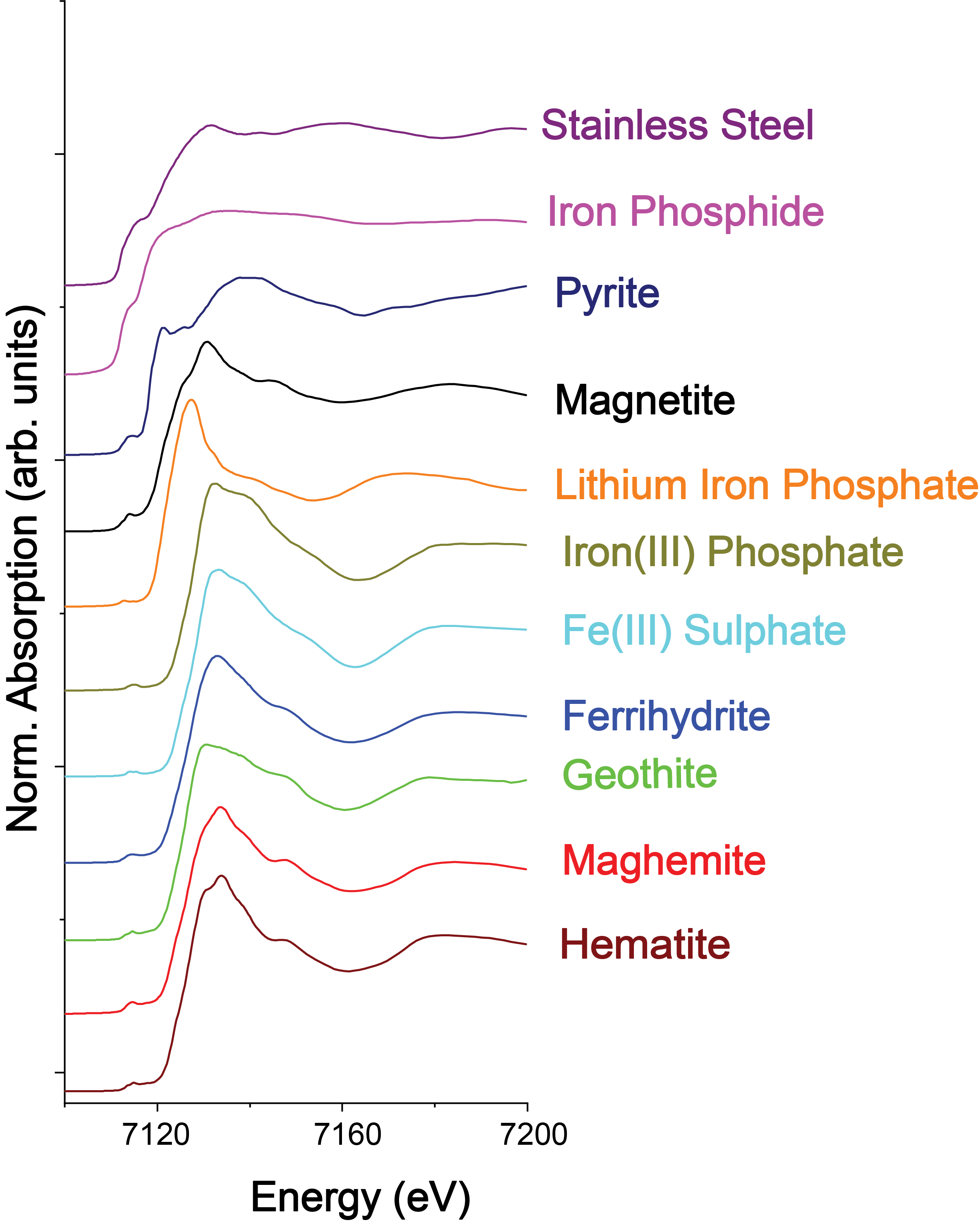


Figure S9. Reference spectra used for combinatorial 2D-XANES fitting.

A. B.


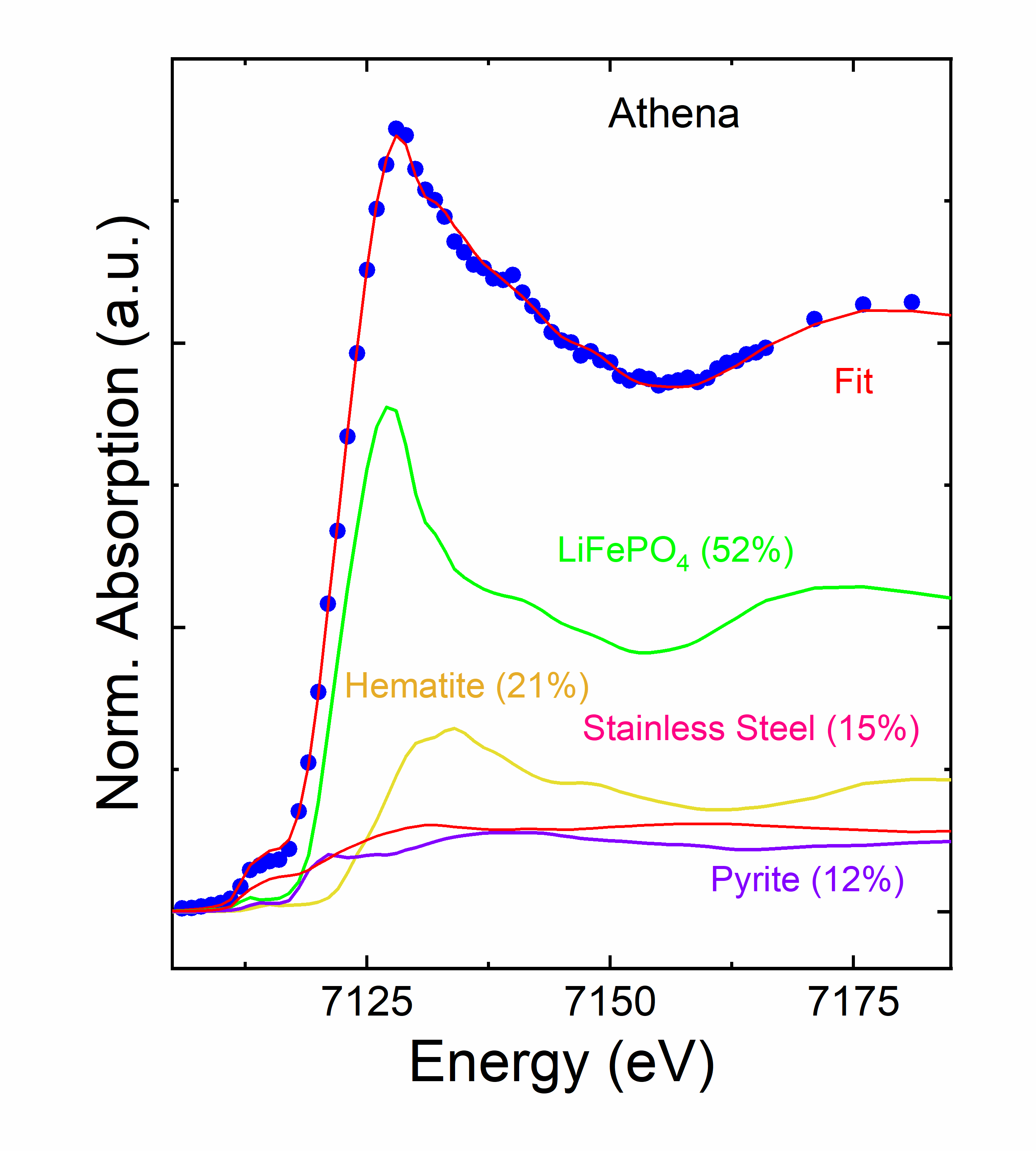

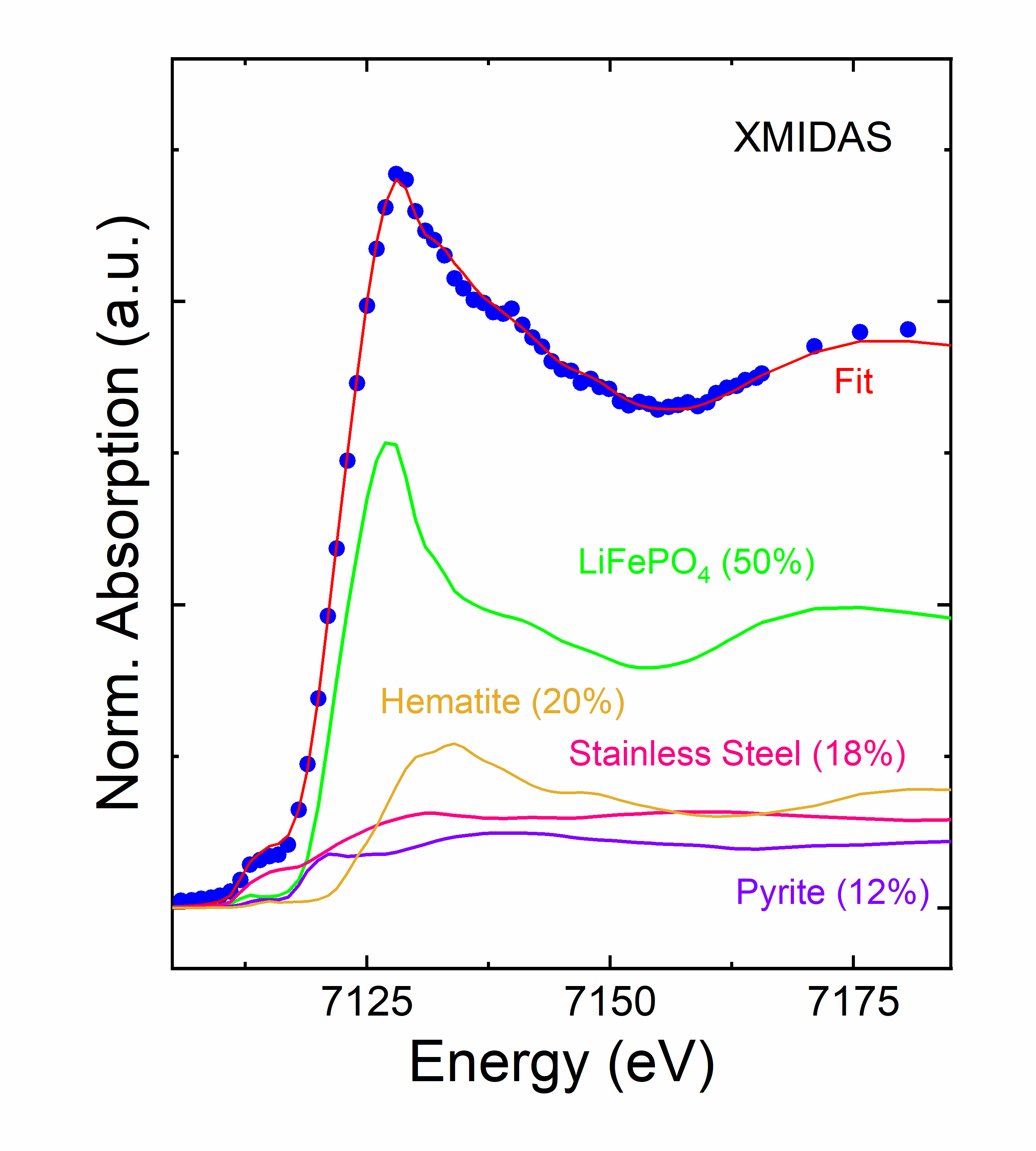


Figure S10. Comparison of XANES fitting results from Athena (A) and XMIDAS (B) programs show similar results


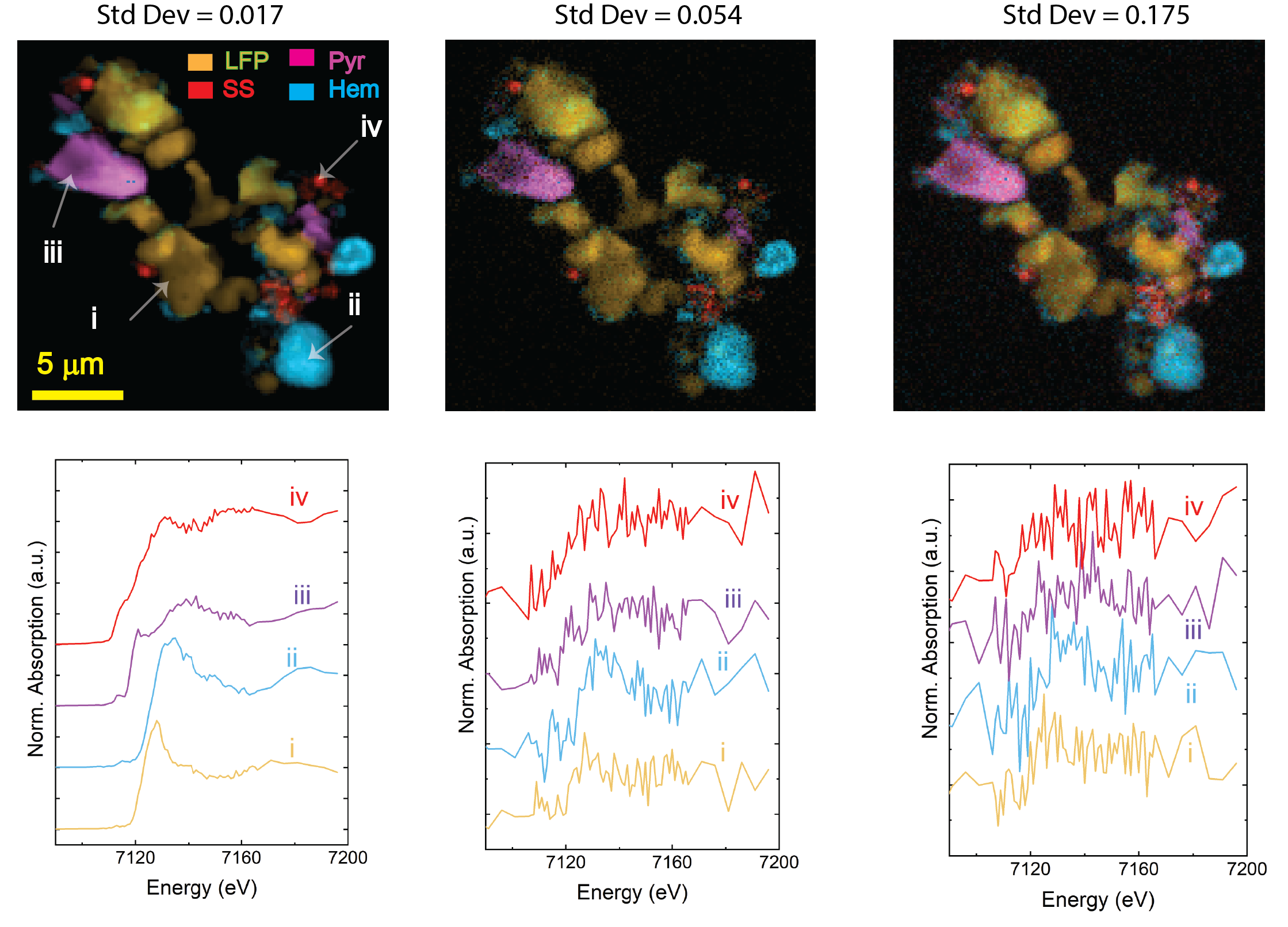


Figure S11. Effect of noise in chemical state analysis. Chemical state maps after introducing random Gaussian noise to the original data (left) to yield 3X (middle) and 10X (right) average standard deviation in the spectrum. Although the chemical state maps using the reference standards showed similar results to the original dataset, the component analysis could not resolve individual components due to noise.


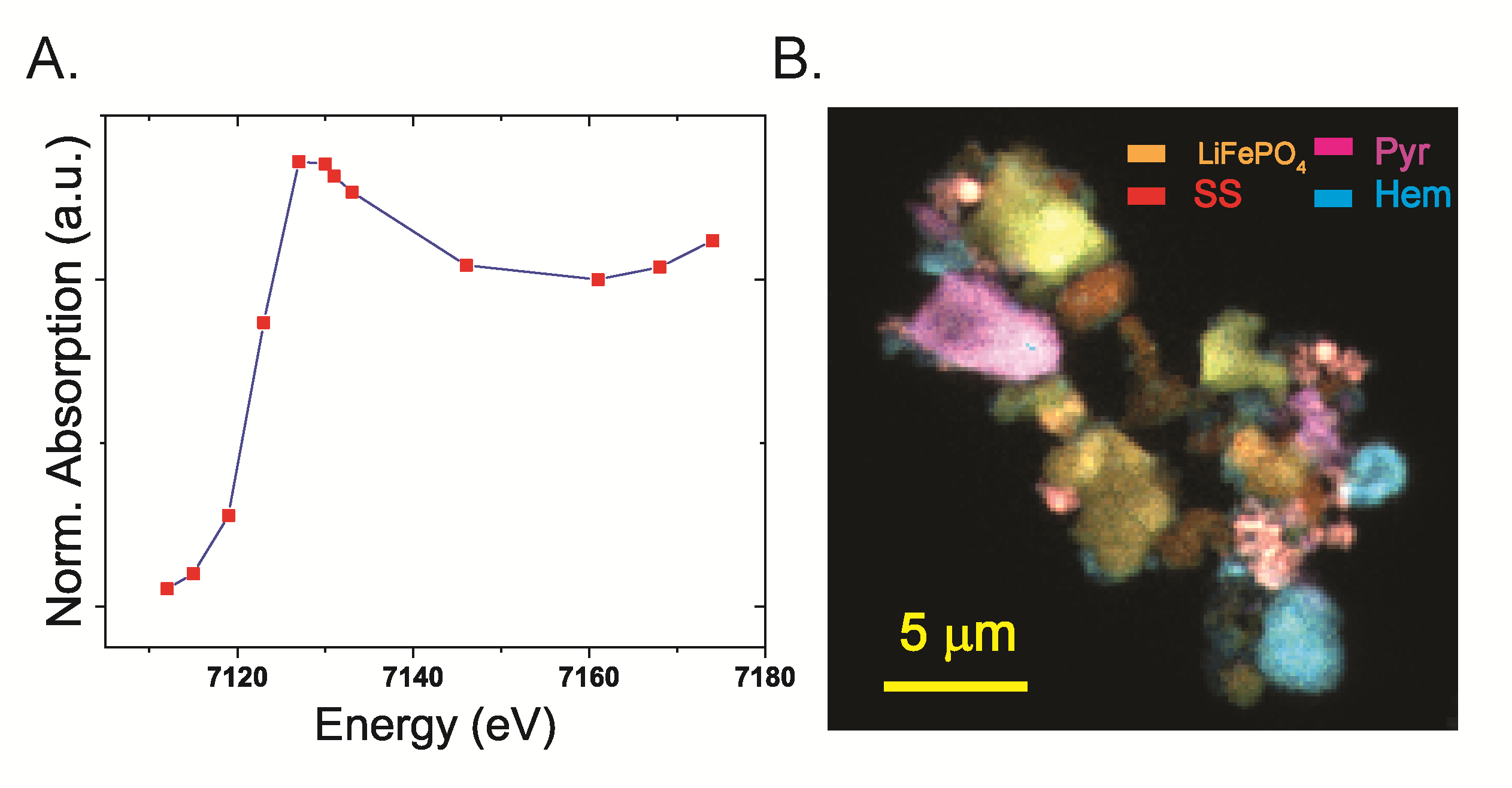


Figure S12. A. Multi-energy point spectrum (12 energies) derived from the nano-XANES data. The energy points are selected based on the peaks and valleys of the reference standard spectra shown in Figure S9. B. The NMF analysis of the reduced data showed similar results as the original data (Figure 4).


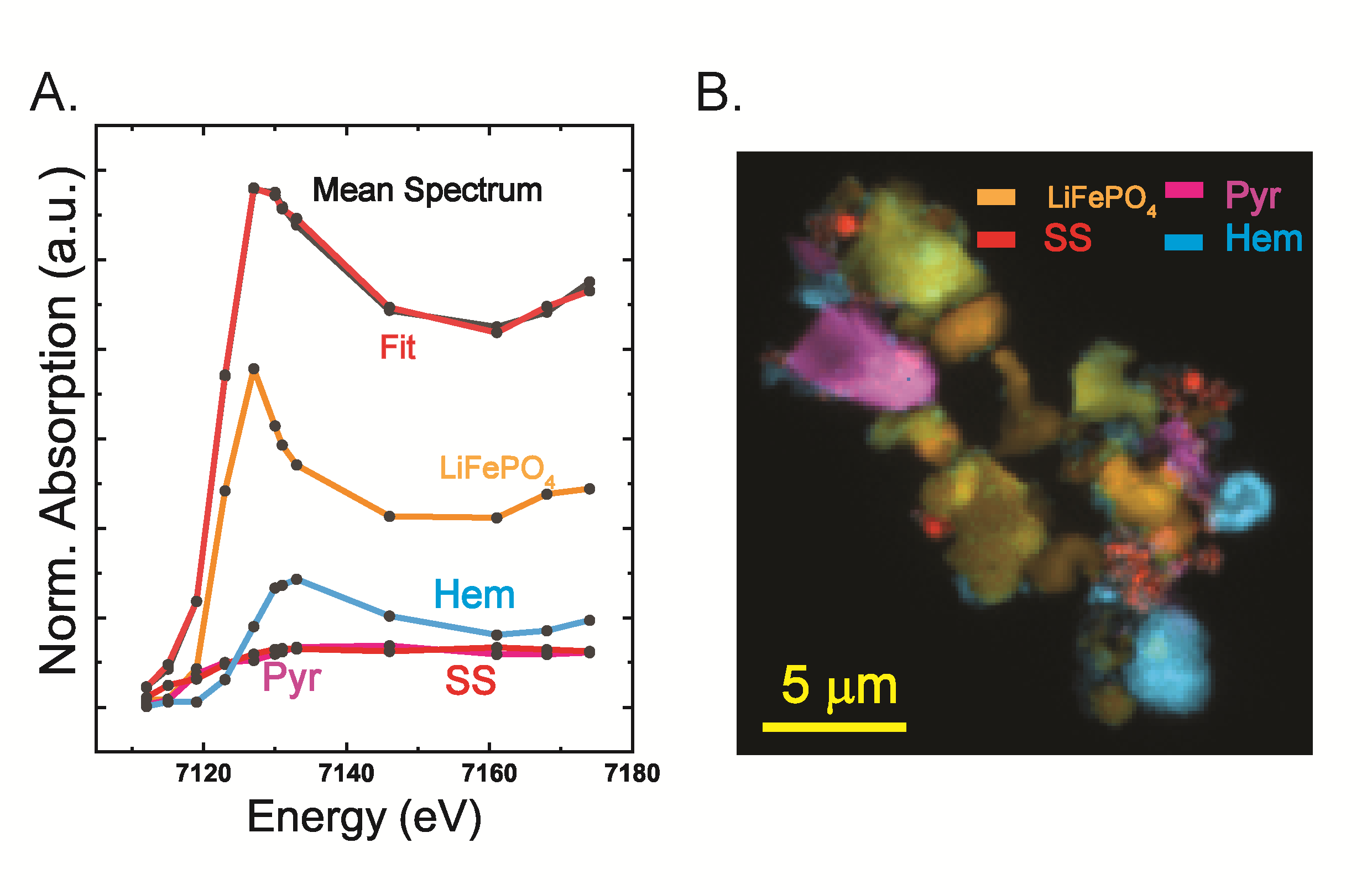


Figure S13. A. Linear combination fitting results of the mean spectrum from multi-energy point data (Figure S12). B. Chemical state map showing similar result to the complete spectrum data (see Table 1 and Table S2). Note that the number of energy points in the reference spectra are also reduced to match the points in the data.

Table S1. Combinatorial XANES fitting results of multi-energy pint maps shown in Figure S12 and S13 (SS = Stainless Steel, Hem = Hematite, Pyr = Pyrite)

| **Name** | **Set of Reference Standards** | **The normalized coefficient for each component** | **r-factor** | **reduced-chi^2^** |
| --- | --- | --- | --- | --- |
| (i) | [LiFePO_4_, SS, Pyr, Hem] | [0.50, 0.18, 0.10, 0.22] | 4.00x10^-5^ | 0.003816 |
| (ii) | [Magnetite, LiFePO_4_, SS, Pyr, Hem] | [0.02, 0.50, 0.18, 0.09, 0.21] | 4.00x10^-5^ | 0.004263 |
| (iii) | [Goethite, LiFePO_4_, SS, Pyr, Hem] | [0.03, 0.51, 0.18, 0.09, 0.19] | 3.70x10^-5^ | 0.004264 |
| (iv) | [Fe_2_(SO_4_)_3_, LiFePO_4_, SS, Pyr, Hem] | [0.03, 0.52, 0.17, 0.1, 0.18] | 3.90x10^-5^ | 0.004274 |
| (v) | [LiFePO_4_, FePO_4_, SS, Pyr, Hem] | [0.51, 0.01, 0.18, 0.1, 0.2] | 4.00x10^-5^ | 0.004318 |

1. O’Day PA, Rivera Jr N, Root R, Carroll SA. X-ray absorption spectroscopic study of Fe reference compounds for the analysis of natural sediments. *American Mineralogist* 2004;**89**:572-85

2. Pattammattel A, Leppert VJ, Aronstein P, Robinson M, Mousavi A, Sioutas C, Forman HJ, O'Day PA. Iron speciation in particulate matter (PM2. 5) from urban Los Angeles using spectro-microscopy methods. *Atmos. Environ.* 2021;**245**:117988
